# TiAl_3_-TiN Composite Nanoparticles Produced by Hydrogen Plasma-Metal Reaction: Synthesis, Passivation, and Characterization

**DOI:** 10.3390/nano6060101

**Published:** 2016-06-01

**Authors:** Ju Ying Li, Qing Song Mei

**Affiliations:** 1 School of Mechanical Engineering, Wuhan Polytechnic University, Wuhan 430023, China; jylimei@163.com; 2 Department of Materials Engineering, School of Power and Mechanical Engineering, Wuhan University, Wuhan 430072, China

**Keywords:** nanoparticles, TiAl_3_, TiN, hydrogen plasma-metal reaction, passivation

## Abstract

TiAl_3_ and TiN composite nanoparticles were continuously synthesized from Ti–48Al master alloy by hydrogen plasma-metal reaction in a N_2_, H_2_ and Ar atmosphere. The phase, morphology, and size of the nanoparticles were studied by X-ray diffraction (XRD) and transmission electronic microscopy (TEM). X-ray photoelectron spectroscopy (XPS) and evolved gas analysis (EGA) were used to analyze the surface phase constitution and oxygen content of the nanoparticles. The as-synthesized nanopowders were mainly composed of nearly spherical TiAl_3_ and tetragonal TiN phases, with a mean diameter of ~42 nm and mass fractions of 49.1% and 24.3%, respectively. Passivation in the atmosphere of Ar and O_2_ for 24 h at room temperature led to the formation of amorphous Al_2_O_3_ shells on the TiAl_3_ particle surface, with a mean thickness of ~5.0 nm and a mass fraction of ~23.5%, as well as TiO_2_ with a mass fraction of ~3.2%.

## 1. Introduction

Hydrogen plasma-metal reaction (HPMR) is an effective method to synthesis nanoparticles of pure metals or alloys that was first developed by Uda and coauthors [1,2,3]. After that, improvements have been made on this method so that metal nanoparticles can be continuously produced [4]. The merits of HPMR include: (1) high generation rate; (2) wide applicability; (3) high purity of the produced particles; and (4) nanoscale particle size. By now, nanoparticles of pure metals and different binary or ternary alloys have been successfully produced by HPMR [5,6,7,8,9]. Synthesis of nanoparticles in a large quantity is important for the manufacturing of components by the traditional powder metallurgy method and the 3D rapid prototyping that has quickly developed in recent years [10]. The Ti-Al system is important for applications in automobile and aerospace industries. In previous studies [11,12], nanoparticles of titanium aluminides were investigated in the Ti-Al binary alloy by HPMR. However, the synthesis of nanopowders containing Ti-Al intermetallic and ceramic nanoparticles has not been reported. Due to the large surface area, the as-synthesized metallic nanoparticles usually need to be passivated before full exposure to the air. Passivation of metallic nanoparticles (e.g., Al nanoparticles) was usually conducted in Ar and O_2_ atmosphere, and sometimes in different liquid or solid substances [13,14]. As passivation can lead to changes in the composition, phase, and property of the surface of nanoparticles, the investigation and characterization of the surface of passivated nanoparticles are necessary [15,16,17] but quantitative characterization is usually difficult. In our previous study [18], Al_2_O_3_/Ti_2_AlN composites with a novel combination of high temperature properties were fabricated successfully from TiAl_3_-TiN composite nanoparticles by HPMR, for which quantitative characterization of the composition, surface structure, and phase fraction of the composite nanoparticles after passivation is of great importance.

In this study, we reported the synthesis and quantitative characterization of TiAl_3_-TiN composite nanoparticles from a Ti-Al binary system by hydrogen plasma-metal reaction in a N_2_, H_2,_ and Ar atmosphere. The phase, morphology, and size of the composite nanoparticles, as well as their passivation behaviors, were studied by X-ray diffraction (XRD), transmission electronic microscopy (TEM), X-ray photoelectron spectroscopy (XPS), and evolved gas analysis (EGA). The surface composition, structure, and phase fraction of the composite nanoparticles after passivation were determined.

## 2. Experiment Procedure

### 2.1. Synthesis of Nanoparticles

The master alloys used in this work were prepared from 99.5% sponge Ti and 99.8% Al buttons by melting three times using a consumable electrode vacuum furnace. The master alloy was designed as Ti–48Al (at. %, the same for below) and machined into 20 mm in diameter and 200 mm in height. The HPMR equipment used in this study was developed on the base of [1,2,3]. The chamber was then evacuated to about 100 Pa using a rotary pump, washed three times with argon gas, and backfilled with high purity argon, hydrogen, and nitrogen to a predetermined pressure. The chamber atmosphere is the mixture gas of N_2_, H_2_, and Ar (0.2:1:1) with a total pressure of 0.1 MPa. The master alloy underwent evaporation, reaction, and condensation to form nanoparticles. The nanoparticles were then transferred by a circulating pump and deposited onto the inner surfaces of the collection chamber. Passivation of nanoparticles was performed in the atmosphere of Ar and O_2_ at room temperature for 24 h.

### 2.2. Characterization

XPS analysis was performed on the ESCALAD-250 spectrometer (Thermo Electron, Waltham, MA, USA) using monochromated Al Kα X-rays (1486.6 eV) and a hemispherical analyzer. The samples were mounted onto carbon adhesive tape. The operating parameters were as follows: the system base pressure was 1 × 10^−6^–7 × 10^−6^ Pa; the diameter of the X-ray beam was 100 μm, and the angle of emission of the detected photoelectrons (relative to the surface normal) was 45°. The evolved gas analysis (EGA) was performed on a TC-436 Oxygen/Nitrogen determinator (LECO, Saint Joseph, MI, USA) operating in the inert gas fusion principle. The samples (0.1–0.3 g in mass) were mixed with a pre-degassed graphite powder as a reducing agent, placed into pre-degassed graphite crucibles, and ramp heated under a helium flow. The evolution of the CO and CO_2_ gases was monitored on-line with non-dispersive infrared detectors (NDIR) (LECO, Saint Joseph, MI, USA). XRD was performed with an Ultima IV diffractometer (Rigaku, Tokyo, Japan) using Cu K_α_ radiation. The microscopic images of the nanoparticles were obtained using a JEOL-2000FX (JEOL, Tokyo, Japan) TEM. High resolution transmission electron microscopy (HRTEM) was conducted on a JEOL-TEM2100 transmission electron microscope (JEOL, Tokyo, Japan). The sample was sonicated in acetone and dropped onto a carbon coated copper grid.

## 3. Results and Discussion

**Figure 1:**
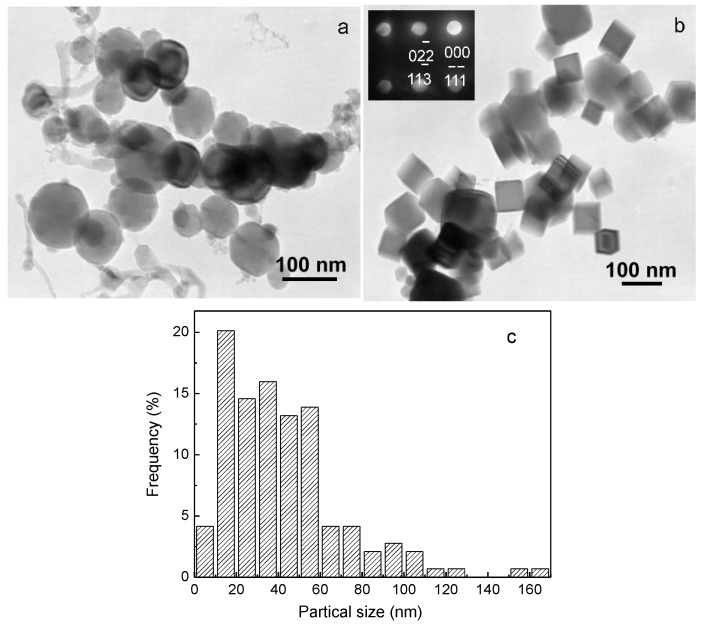
(**a**) and (**b**) Typical bright-field transmission electron microscope (TEM) micrographs of the nanoparticles synthesized from the master alloy of Ti–48Al by hydrogen plasma-metal reaction (HPMR) in N_2_, H_2_, and Ar atmosphere; (**c**) particle size distribution.

**Figure 2:**
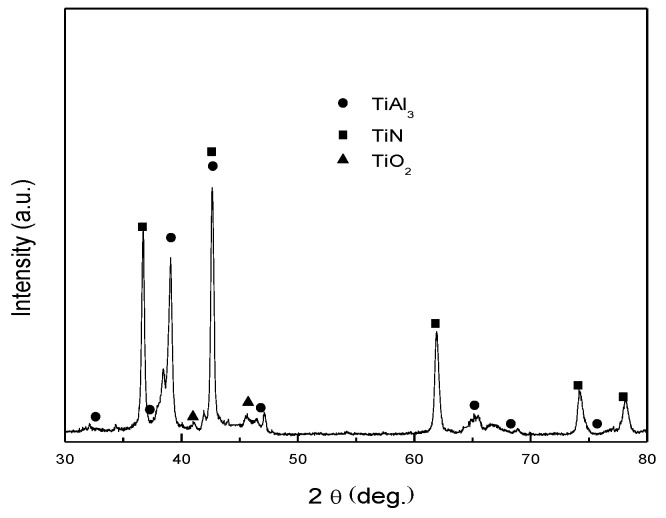
X-ray diffraction analysis (XRD) pattern of the nanoparticles synthesized from the master alloy of Ti–48Al by HPMR in N_2_, H_2_, and Ar atmosphere.

Figure 1 shows typical bright-field TEM images of nanoparticles produced from Ti–48Al master alloy in the N_2_, H_2_, and Ar atmosphere. Two kinds of morphologies of the as-synthesized nanoparticles can be seen from Figure 1: nearly spherical and tetragonal shapes, all dispersing well on the carbon film. The inset of Figure 1b is the corresponding micro-diffraction pattern of the tetragonal particle, which can be indexed as TiN phase. At high temperatures produced by the electric arc, nitrogen atoms can react with the metal vapor containing two elements: Ti and Al. Here TiN phase is formed instead of AlN by the selective reaction of N and Ti atoms. Thermodynamic analysis can explain this selectivity: at the same temperature, the enthalpy of formation of TiN is lower than that of AlN (e.g., the values of enthalpy of formation of TiN and AlN at 298 K are −339.4 kJ/mol and −319.2 kJ/mol, respectively, and those at 2300 K are −264.9 kJ/mol and −258.6 kJ/mol, respectively) [19]. Figure 1c is the particle size distribution of as-synthesized nanoparticles. As shown in Figure 1c, the distribution of nanoparticle size is between 10 nm and 200 nm, with an average of about 42 nm. Interestingly, although the as-synthesized nanoparticles contain two main phases with different morphologies, the average sizes of them are similar. The XRD pattern (Figure 2) further indicates that the as-synthesized nanoparticles comprise two main phases of TiAl_3_ and TiN. This is different from the Ti-Al nanoparticles synthesized in Ar and H_2_ atmosphere: the Ti-Al nanoparticles synthesized in Ar and H_2_ atmosphere from the same master alloy are composed of TiAl_3_, Ti_2_Al_5_, TiAl, and Al phases [11]. In this study, TiAl and Ti_2_Al_5_ phases did not appear as indicated by XRD. This is because in the nitrogen-containing atmosphere, Ti first reacts with N to form TiN, which consumes a large fraction of Ti. As Ti content in the vapor decreases, the formation of TiAl and Ti_2_Al_5_ phases is suppressed.

**Figure 3:**
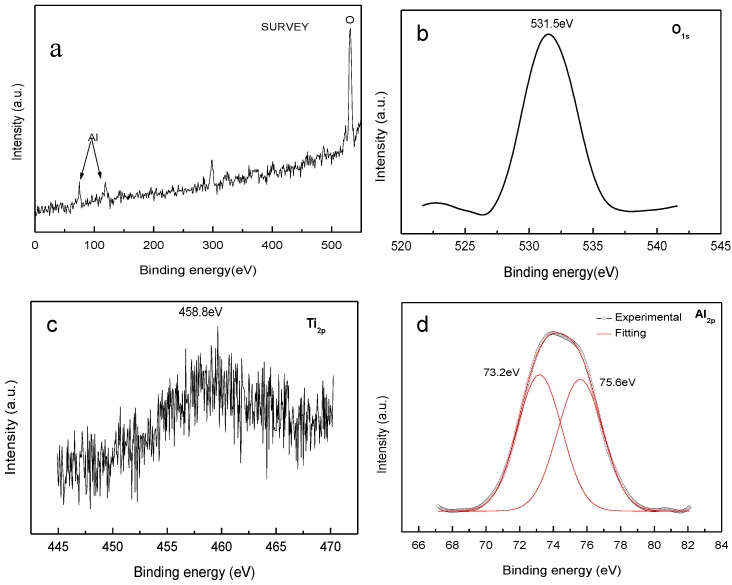
(**a**) X-ray photoelectron spectroscopy (XPS) patterns of the nanoparticles synthesized from the master alloy of Ti–48Al by HPMR in N_2_, H_2_, and Ar atmosphere; (**b**–**d**) are the enlargements corresponding to different ranges of binding energy.

Due to the large surface area and high surface activity, the composite nanoparticles were passivated to avoid violent reaction with oxygen by direct exposure to air. Figure 3 is the XPS result of the composite nanoparticles after passivation. As shown in Figure 3, the surface elements of nanopowders are mainly composed of Al, O, and Ti. As can be seen from Figure 3b,c, the O_1S_ photoelectron spectrum shows the binding energy of 531.5 eV, and the Ti_2p_ photoelectron spectrum shows the binding energy of 458.8 eV. As shown in Figure 3d, the fitting analysis of the Al_2p_ photoelectron spectrum agrees well with the experiment data, indicating the binding energies of 73.2 eV and 75.6 eV, respectively. Compared with the standard database, the state of Al element is 3+ of Al_2_O_3_, and the state of Ti element is 4+ of TiO_2_ [20]. A semi quantitative analysis of the surface elemental composition of the composite nanoparticles is listed in Table 1. Table 1 generally indicates that the main surface phase of nanopowders is Al_2_O_3_, with only a small amount of TiO_2_.

**Figure 4:**
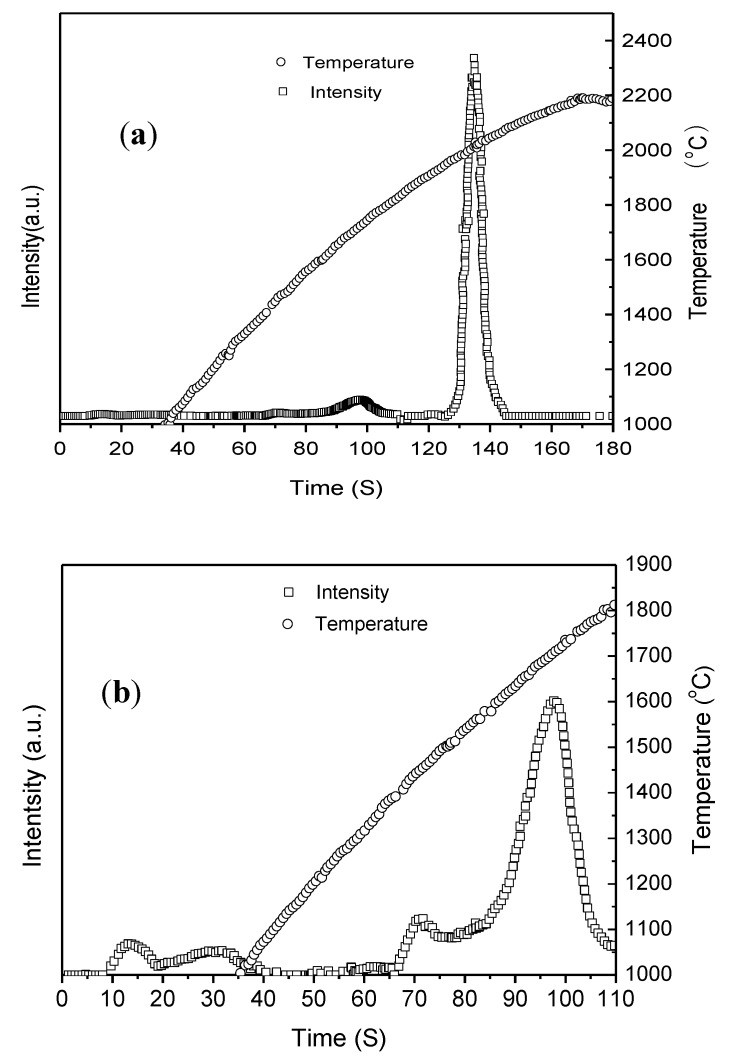
(**a**) Release curve of oxygen in the passivated nanopowders synthesized from the master alloy of Ti–48Al by HPMR in N_2_, H_2_, and Ar atmosphere; (**b**) the amplification in the time range of 0–110 s.

Figure 4 shows the total oxygen release curve of the passivated nanoparticles. As shown in Figure 4a, the main oxygen release peak occurs at the time between 110 s and 152 s, and the temperature between 1900 °C and 2100 °C, which corresponds to the oxygen release peak of Al_2_O_3_ phase in the nanopowders. The corresponding oxygen fraction for this peak is 12.22 wt. %. As shown in Figure 4b, the oxygen release peak at the time between 66 s and 110 s and the temperature between 1400 °C and 1800 °C has the oxygen content of 1.30 wt. %. It can be concluded that this is the oxygen release peak for TiO_2_ in the nanopowders. In Figure 4b, the oxygen release peak at the time between 0 s and 41 s and the temperature between 2200 °C and 1100 °C has the oxygen content of 0.18 wt. %, which corresponds to the release peak of adsorbed oxygen. The above results indicate that oxygen in the nanopowders mainly exists in the form of Al_2_O_3_ compounds, with a small amount in TiO_2_ compounds and surface adsorbed oxygen.

The mass fraction of TiN (W_TiN_) in the powder can be calculated by:

W_TiN_ = W_N_ + W_Ti/TiN_ (1)

where W_N_ is the mass fraction of N in the powder, and W_Ti/TiN_ is the mass fraction of Ti in TiN, which can be calculated by:

W_Ti/TiN_ = W_N_(M_Ti_ + M_N_)/M_N_ (2)

where M_Ti_ and M_N_ are the atomic mass of Ti and N, respectively. The mass fraction of TiO_2_ (W_TiO_2__) in the powder can be calculated by:

W_TiO_2__ = W_O/TiO_2__ + W_Ti/O_2__ (3)

where W_O/TiO_2__ and W_Ti/TiO_2__ are the mass fraction of O and Ti in TiO_2_, respectively. W_Ti/TiO_2__ can be calculated by:

W_Ti/TiO_2__ = W_O/TiO_2__M_Ti_/2M_O_ (4)

where M_O_ is the atomic mass of oxygen.

Similarly, the mass fraction of Al_2_O_3_ (W_Al_2_O_3__) in the powder can be calculated by:

W_Al_2_O_3__ = W_O/Al_2_O_3__(1 + 2M_Al_/3M_O_) (5)

where M_Al_ is the atomic mass of Al. Also, the mass fraction of TiAl_3_ (W_TiAl_3__) can be calculated by:

W_TiAl_3__ = (W_Ti_ − W_Ti/TiN_ − W_Ti/TiO_2__) × (1 + 3M_Al_/M_Ti_) (6)

where W_Ti_ is the mass fraction of Ti in the powder.

Table 2 lists the elemental composition of the nanopowders after passivation, measured by gas analysis and chemical analysis, as well as the fractions of oxygen in Al_2_O_3_, TiO_2_, and adsorbed oxygen. Using the data in Table 2, the mass fraction of difference phases in the nanopowders can be calculated by Equations (1)–(6), and the results are given in Table 3.

**Figure 5:**
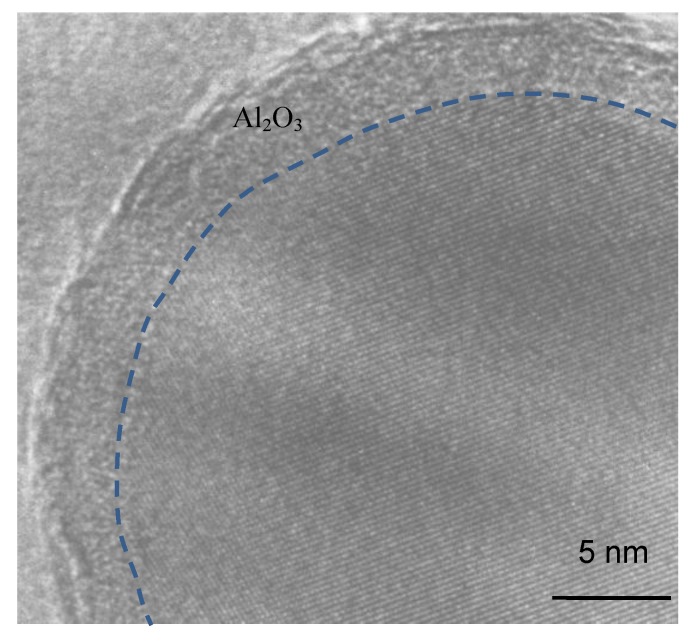
High resolution transmission electron microscopy (HRTEM) micrograph of the passivated TiAl_3_ nanoparticle showing the surface oxide.

As shown in Table 3, Al_2_O_3_ has a mass fraction of about 23.5% in the nanopowders. However, the XRD pattern of the nanopowders (Figure 2) does not show the Al_2_O_3_ peak. This is because the surface oxide is an amorphous structure, as shown in Figure 5. As shown in Figure 5, Al_2_O_3_ forms a thin shell structure on the surface of the TiAl_3_ particle, as similar to the surface oxide shells of Al and Fe nanoparticles [16,17]. Here, the average shell thickness (*t*) can be estimated by:

(7) $$t=\left(\sqrt[{3}]{1+\frac{W_{\text{Al}2O3}\rho_{\text{TiAl}3}}{W_{\text{TiAl}3}\rho_{\text{Al}2O3}}}-1\right)R$$

where *R* is the radius of the TiAl_3_ particle, and ρ_Al_2_O_3__ and ρ_TiAl_3__ are the density of TiAl_3_ and Al_2_O_3_, respectively. From Equation (7), an average shell thickness of about 5.0 nm is obtained, which agrees well with the HRTEM observations (Figure 5). The surface oxide Al_2_O_3_ is dense and protective, which can hinder the further oxidation of nanopowders in air. No further increase of the oxygen content was found in the sample of nanopowders after exposed in air for more than 600 h.

## 4. Conclusions

In this study, nanopowders were produced continuously by the HPMR method in a N_2_, H_2_ and Ar atmosphere from the master alloy of Ti–48Al, followed by passivation in the Ar and O_2_ atmosphere for 24 h at room temperature. The phase constitution, morphology, and size of the nanopowders were investigated, and the surface composition and phase were quantitatively characterized. The main results are summarized as follows:

(1) The nanopowders are mainly composed of TiAl_3_ and TiN phases with an average diameter of ~42 nm. TiAl_3_ nanoparticles are nearly spherical with a mass fraction of ~49.1%, and TiN nanoparticles are tetragonal with a mass fraction of ~24.3%.
(2) Passivation of the nanopowders led to the formation of protective amorphous Al_2_O_3_ shells on the particle surface, with a mean thickness of ~5.0 nm and a mass fraction of ~23.5%, as well as TiO_2_ with a mass fraction of ~3.2%.

## Figures and Tables

**Table 1:** Surface composition of the nanopowders.

Element	O	Al	Ti
Content (at. %)	60.9%	38.4%	1.7%

**Table 2:** Mass fractions of the elements in the nanopowders and those of oxygen in different phases. See the text for details.

W_N_	W_Ti_	W_Al_	W_O_		
			W_OA_	W_O/Al_2_O_3__	W_O/TiO_2__
5.5%	39.0%	41.8%	0.18%	12.22%	1.3%

**Table 3:** Calculated mass fractions of different phases in the nanopowders. See the text for details.

W_TiN_	W_TiAl_3__	W_Al_2_O_3__	W_TiO_2__
24.3%	49.1%	23.5%	3.2%
